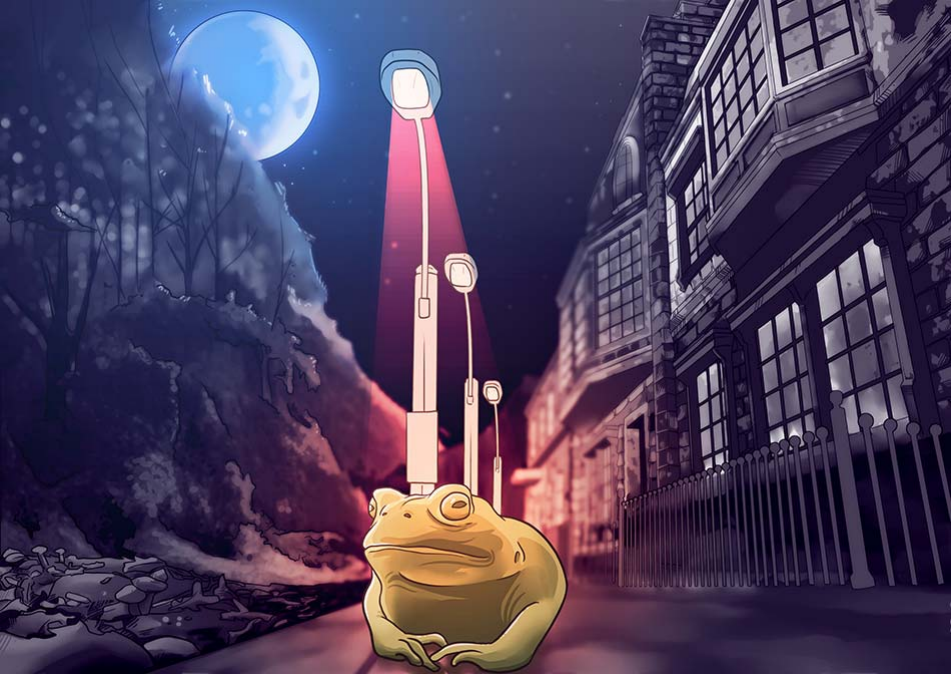# Dark matters: night light stops toads in their tracks

**DOI:** 10.1093/conphys/coz085

**Published:** 2019-11-19

**Authors:** Alison Haynes

**Affiliations:** Centre for Sustainable Ecosystem Solutions, University of Wollongong, Wollongong, NSW 2522, Australia

Humans like night lights because, although we do not see so well in the dark, we do like to stay up past sunset. But how do night lights affect other species, particularly nocturnal species? As a new study shows, if you are a common toad, even the glow from a street light could mean you are not as active as you should be at night. To make matters worse, night lights may also make toads stressed, which can have dire consequences for survival. And, as it happens, this kind of response may be universal among vertebrates, from fishes to gorillas.

With nearly 25% of the Earth’s surface now lit up at night, a figure that is rising by 6% every year, it is becoming more and more important to understand the effects of artificial light at night (ALAN), as Morgane Touzot from the University of Lyon and her colleagues from France would agree. She and her team investigated the effects of ALAN on the common toad (*Bufo bufo*). This toad is widely distributed in forests and bushlands across Europe and parts of Northern Asia and North Africa. Why toads? Well, amphibians are good to study in general, as there are a lot of nocturnal species; moreover, amphibians are among the most threatened groups of animals on Earth. Understanding stressors that pose a risk to their survival is imperative.

Touzot and her team caught 36 male wild toads at the beginning of the breeding season and subjected them to a variety of light levels for 20 days while monitoring their behaviour and oxygen uptake. One light level mimicked moonlight (0.1 lux) and acted as a control. Another light level was representative of a typical street light (5 lux), while a third, at 20 lux, mimicked urban park lights.

The team found that, for toads exposed to artificial light, their activity was down by as much as 73%. In addition, artificial light triggered a reallocation of energy. Toads exposed to artificial light were likely spending more energy on maintenance costs and, as a result, had to reduce activity. Perhaps this added maintenance cost was to cope with stress.

Touzot’s results are concerning. With current, rapid urbanization, a lot of naturally dark habitats are being lost. And, it is not just streets and cities that are lit up at night. Restored areas, many of which contain wetlands that are important habitat for a lot of species, are experiencing artificial light, making ALAN an emerging stressor that puts many species at risk.

Although many solutions have been proposed, few have been thoroughly tested. For instance, we could maintain dark areas, change spectra and minimize the blue wavelength that is particularly disruptive to circadian rhythms. We could also dim lights or switch lights on for only part of the night. Conservation physiology has told us about the dark side of night light. It is encouraging that we can glimpse potential solutions, but now it is time to start evaluating just how those solutions might impact our ecosystems so we can help our wildlife reclaim the night.

Section Editor**:** Jodie L. Rummer

Illustration by Erin Walsh; Email: ewalsh.sci@gmail.com

**Figure:**